# *Proteus mirabilis* from Captive Giant Pandas and Red Pandas Carries Diverse Antimicrobial Resistance Genes and Virulence Genes Associated with Mobile Genetic Elements

**DOI:** 10.3390/microorganisms13081802

**Published:** 2025-08-01

**Authors:** Yizhou Yang, Yan Liu, Jiali Wang, Caiwu Li, Ruihu Wu, Jialiang Xin, Xue Yang, Haohong Zheng, Zhijun Zhong, Hualin Fu, Ziyao Zhou, Haifeng Liu, Guangneng Peng

**Affiliations:** 1 Agricultural Animal Diseases and Veterinary Public Health Key Laboratory of Sichuan Province, College of Veterinary Medicine, Sichuan Agricultural University, Chengdu 611130, China; yangyizhou1201@163.com (Y.Y.); wangjiali1113@163.com (J.W.); 18728186121@163.com (R.W.); xinjialiang123@163.com (J.X.); yangx00112@163.com (X.Y.); haohongzheng916@gmail.com (H.Z.); zhongzhijun488@126.com (Z.Z.); fuhl.sicau@163.com (H.F.); zzhou@sicau.edu.cn (Z.Z.); 410140017@163.com (H.L.); 2 Beijing Key Laboratory of Captive Wildlife Technologies, Beijing Zoo, Beijing 100044, China; liuyanllh@sohu.com; 3 China Conservation and Research Center for the Giant Panda, Key Laboratory of State Forestry and Grassland Administration on the Giant Panda, Chengdu 610066, China; licaiwu118@163.com

**Keywords:** captive pandas, *Proteus mirabilis*, antimicrobial resistance, virulence-associated genes, mobile genetic elements

## Abstract

*Proteus mirabilis* is a zoonotic pathogen that poses a growing threat to both animal and human health due to rising antimicrobial resistance (AMR). It is widely found in animals, including China’s nationally protected captive giant and red pandas. This study isolated *Proteus mirabilis* from panda feces to assess AMR and virulence traits, and used whole-genome sequencing (WGS) to evaluate the spread of resistance genes (ARGs) and virulence genes (VAGs). In this study, 37 isolates were obtained, 20 from red pandas and 17 from giant pandas. Multidrug-resistant (MDR) strains were present in both hosts. Giant panda isolates showed the highest resistance to ampicillin and cefazolin (58.8%), while red panda isolates were most resistant to trimethoprim/sulfamethoxazole (65%) and imipenem (55%). Giant panda-derived strains also exhibited stronger biofilm formation and swarming motility. WGS identified 31 ARGs and 73 VAGs, many linked to mobile genetic elements (MGEs) such as plasmids, integrons, and ICEs. In addition, we found frequent co-localization of drug resistance genes/VAGs with MGEs, indicating a high possibility of horizontal gene transfer (HGT). This study provides crucial insights into AMR and virulence risks in *P. mirabilis* from captive pandas, supporting targeted surveillance and control strategies.

## 1. Introduction

*Proteus mirabilis* is a commensal bacterium commonly found in the gastrointestinal tracts of humans and animals, yet it is also recognized as an opportunistic pathogen capable of causing a broad spectrum of infections [1]. *P. mirabilis*-associated diseases, including acute diarrhea, reproductive tract infections, and wound infections, have been documented in both giant pandas and red pandas [2]. This pathogen carries multiple virulence factors that enhance colonization and pathogenic potential: fimbriae mediate epithelial adhesion; urease contributes to the formation of struvite and calcium phosphate urinary stones; flagella facilitate motility; and cytotoxicity is enhanced by both hemolysin and the secreted protease ZapA [3]. Furthermore, *P. mirabilis* exhibits unique swimming and swarming motility, enabling rapid dissemination within the host [4]. It is also capable of forming crystalline biofilms, substantially increasing its resistance to antimicrobial agents [5]. In recent years, antimicrobial resistance (AMR) in *P. mirabilis* has become an escalating concern [6]. Under antimicrobial selective pressure, *P. mirabilis* continuously acquires additional antimicrobial resistance genes (ARGs) via horizontal gene transfer (HGT), contributing to the emergence of multidrug-resistant (MDR) strains. In recent years, extensively drug-resistant (XDR) and even pandrug-resistant (PDR) *P. mirabilis* isolates have been reported, and strains producing ESBLs and carbapenemases have been widely detected in animals [7]. Combined with its intrinsic resistance to polymyxins, nitrofurantoin, tigecycline, and tetracyclines, treatment options for *P. mirabilis* infections have become increasingly limited [8].

The giant panda (*Ailuropoda melanoleuca*) and red panda (*Ailurus fulgens*), two endangered mammalian species endemic to the Himalaya–Hengduan Mountains region, hold significant research value in evolutionary biology and conservation medicine [4,9]. Although they belong to distinct families within the order Carnivora (*Ursidae* vs. *Ailuridae*), both species exhibit highly specialized bamboo-feeding adaptations and overlapping ecological niches as a result of convergent evolution [10]. This ecological convergence has led to widespread zoo management practices that house both species in shared enclosures, based on habitat similarity. Given that numerous zoos and conservation organizations conduct rescue and reintroduction programs for giant pandas and red pandas, the introduction of highly resistant *P. mirabilis* strains into the wild could pose a serious threat to other wildlife [11]. Additionally, red pandas are often housed in open enclosures, and public interactions for educational purposes may create pathways for direct or indirect transmission of resistant *P. mirabilis* between humans and pandas [12]. Therefore, elucidating the antimicrobial resistance mechanisms and transmission pathways of *P. mirabilis* is a critical step toward mitigating these emerging threats. However, such studies remain scarce, and existing data are insufficient to inform effective control strategies.

In this study, *P. mirabilis* strains were isolated from the feces of both giant and red pandas in various zoos and conservation centers across Sichuan Province. We assessed their antimicrobial resistance profiles and virulence-associated phenotypes. Moreover, through whole-genome sequencing, we conducted a comprehensive genomic analysis to uncover the genetic diversity, antimicrobial resistance genes, and virulence determinants among *P. mirabilis* isolates from different geographic locations and host species (giant pandas and red pandas). This study aims to characterize the antimicrobial resistance and pathogenic potential of *P. mirabilis* in the gut microbiota of captive giant and red pandas, thereby providing a scientific basis for future surveillance, targeted control strategies, and conservation practices.

## 2. Materials and Methods

### 2.1. Sample Collection, Identification

Fresh fecal samples were collected from giant pandas (*Ailuropoda melanoleuca*) and red pandas (*Ailurus fulgens*) housed in zoos and Giant Panda Conservation and Research Centers located in Chengdu, Dujiangyan, Ya’an, and Wenchuan, Sichuan Province, China, between 18 January and 6 July 2024. All samples were immediately frozen and transported to the laboratory under cold chain conditions for the isolation of *Proteus mirabilis*.

Approximately 5 g of each sample was inoculated into Luria–Bertani (LB) broth (Hopebio, Qingdao, China) and incubated at 37 °C with shaking at 120 rpm for 24 h. After incubation, a loopful of the enriched culture was streaked onto LB agar plates and incubated at 37 °C for 24 h. Colonies exhibiting characteristic swarming motility were further subcultured onto Salmonella–Shigella (SS) agar plates (Hopebio, Qingdao, China) and incubated at 37 °C for an additional 24 h. Colonies displaying translucent circular morphology with dark centers were selected for further analysis [13].

Species identification was performed by PCR amplification of the 16S rRNA gene using universal primers 27F (5′-AGAGTTTGATCCTGGCTCAG-3′) and 1492R (5′-TACGGCTACCTTGTTACGACTT-3′) (Sangon Biotech, Shanghai, China) in a 25 μL reaction volume. The PCR conditions were as follows: an initial denaturation at 95 °C for 5 min; followed by 30 cycles of denaturation at 94 °C for 30 s, annealing at 55 °C for 30 s, and extension at 72 °C for 30 s; with a final extension at 72 °C for 7 min. The PCR products were sent to Sangon Biotech (Shanghai, China) for Sanger sequencing, and the resulting sequences were analyzed using BLAST (https://blast.ncbi.nlm.nih.gov/Blast.cgi) against the NCBI nucleotide database. Isolates showing ≥99% sequence identity to *P*. *mirabilis* reference strains ATCC 29906 were identified as *P. mirabilis.*

### 2.2. Antimicrobial Susceptibility Testing

Antimicrobial susceptibility testing was conducted using the broth microdilution method in accordance with the Clinical and Laboratory Standards Institute (CLSI) guidelines (CLSI VET08 and M100, CLSI, Wayne, PA, USA) [14]. Briefly, twofold serial dilutions of each antimicrobial agent (Dalian Meilun Biotech Co., Ltd., Dalian, China) were prepared in cation-adjusted Mueller–Hinton broth (Hopebio, Qingdao, China) in 96-well microtiter plates. Bacterial suspensions were adjusted to a final concentration of approximately 5 × 10^5^ CFU/mL and inoculated into the wells (100 μL per well). The plates were incubated at 37 °C for 18–24 h, and the minimum inhibitory concentrations (MICs) were defined as the lowest concentration of the antibiotic that completely inhibited visible bacterial growth.

The antimicrobials tested included β-lactams (ampicillin, cefazolin, ceftriaxone), aminoglycosides (gentamicin), tetracyclines (tetracycline, doxycycline), sulfonamides (trimethoprim/sulfamethoxazole), quinolones (enrofloxacin, levofloxacin), phenicols (florfenicol), and carbapenems (imipenem, meropenem). MIC results were interpreted according to CLSI VET08 and M100 breakpoints [14]. All tests were performed in triplicate, and *Escherichia coli* ATCC 25922 was used as the quality control strain.

Compared with the disc diffusion method, the broth microdilution method was selected because it provides more accurate and quantitative MIC values. Additionally, *P. mirabilis* exhibits strong swarming motility on agar surfaces, which can lead to overgrowth into the inhibition zones during disc diffusion tests, making zone diameters difficult to interpret reliably.

### 2.3. Phenotypic Assays of Virulence-Associated Characteristics

Biofilm formation was evaluated using the crystal violet staining method in 96-well microtiter plates. The overnight cultures were adjusted to an OD_600_ of 0.1 and subsequently incubated at 37 °C for 24 h. Wells containing only LB broth were used as negative controls. After the incubation period, the wells were washed with PBS, fixed with 200 μL methanol for 15 min, and then the methanol was removed. The wells were stained with a 1% crystal violet solution for 30 min. Excess stain was washed off with sterile water, and the biofilms were solubilized in 33% acetic acid for 30 min. The absorbance was measured at 590 nm. The cut-off OD (ODc) was defined as the mean OD of the negative control plus three times the standard deviation (SD). The strains were classified based on OD thresholds as follows: non-biofilm formers (OD ≤ ODc), weak (ODc < OD_590_ ≤ 2ODc), moderate (2ODc < OD_590_ ≤ 4ODc), strong (4ODc < OD_590_ ≤ 8ODc), and very strong (OD_590_ > 8ODc) biofilm formers based on OD thresholds [15].

The urease activity in *P. mirabilis* was determined using the commercial urease activity assay kit BC4115 (Solarbio, Beijing, China).

The swarming motility was assessed on LB agar plates containing 1.5% agar. Bacterial cultures were grown in LB broth at 37 °C for 12 h and adjusted to an OD_600_ of 0.5. Subsequently, 5 μL of the bacterial suspension was spotted at the center of each plate. The plates were incubated at 37 °C for 9 h in an inverted position. The diameter of the swarming zone was measured and converted to percentage surface coverage. Motility was categorized as weak (≤5%), moderate (5–25%), or strong (>25%) [13].

For hemolysis activity, bacterial strains were inoculated onto 5% defibrinated sheep blood agar plates and incubated at 37 °C for 24 h. The hemolysis was observed and recorded [13].

### 2.4. Whole-Genome Sequencing

Whole-genome sequencing (WGS) was performed on all selected *P. mirabilis* strains in this study. Genomic DNA was extracted from the selected *P. mirabilis* strains using the Quick-DNA Fungal/Bacterial Kit D6005 (Zymo Research, Irvine, CA, USA) according to the manufacturer’s standard protocol. The quality and concentration of the extracted DNA were assessed by electrophoresis on a 1% agarose gel. The DNA concentration was quantified using a NanoDrop2000 spectrophotometer (Thermo Scientific, Waltham, MA, USA) and a Qubit 4 Fluorometer (Thermo Scientific, Waltham, MA, USA). DNA libraries were then prepared using the NEBNext Ultra™ II DNA Library Prep Kit (New England BioLabs, Ipswich, MA, USA) and sequenced on the NovaSeq 6000 platform using the paired-end 2 × 150 bp sequencing protocol (Rhonin Biosciences, Chengdu, China). The genome sequence of the strain isolated in this study has been uploaded to BioProject with the sequence number PRJNA1274410.

### 2.5. Bioinformatic Analysis

Genome assembly was performed using SPAdes v3.12.0 [16]. Gene annotation was conducted with Prokka v1.14.6 [17]. Potential acquired ARGs were identified by ABricate v1.0.1 [18]. Protein sequences were aligned to the Virulence Factors of Pathogenic Bacteria (VFDB) SetB dataset (identity > 90%, E-value cutoff 1 × 10^−5^) using DIAMOND for virulence-associated genes (VAGs) annotation [19]. All annotated assemblies from Prokka were input into Roary v3.13.0 for the generation of both accessory and core genomes [20]. Based on the gene presence–absence matrix generated by Roary, genes that were present in more than 70% of isolates from a specific host or region, but in less than 30% of isolates from other hosts or regions, were defined as host- or region-enriched genes.

Core genome SNPs were identified using Snippy v4.6.0 (https://github.com/tseemann/snippy) accessed on 16 May 2025 with the --ctgs option. A maximum likelihood phylogenetic tree was constructed using RAxML v8.2.12 under the GTRGAMMA model and visualized with ChiPlot [21,22]. Insertion sequences and transposons were predicted using IS Finder [23]. Integrons were predicted using integron_finder v2.0.2 [24]. Integrative and conjugative elements (ICEs) were predicted using the online ICEfinder tool [25]. The online PHASTEST tool was used to predict prophage regions [26]. Plasmid reconstruction, clustering, and typing were performed using MOB-suite v3.1.9 [27]. Boxplots of antimicrobial resistance were generated by host and geographic origin using the R packages tidyverse, ggpubr, and rstatix. Spearman correlation analysis was conducted using readxl, tidyverse, reshape2, and ggplot2. Colocalization analysis was conducted with the R packages tidyverse, igraph, and ggraph, with a colocalization threshold set at 5000 bp. All remaining bioinformatic analyses and visualizations were conducted in R v4.4.2 and RStudio v2024.09.1+394 using the appropriate R packages. All R scripts used in this study are available at https://github.com/Kapid2/Proteus_mirabilis_Analysis (accessed on 13 June 2025).

## 3. Results

### 3.1. Isolation of Proteus mirabilis from Captive Giant and Red Pandas in Sichuan Province

**Figure 1:**
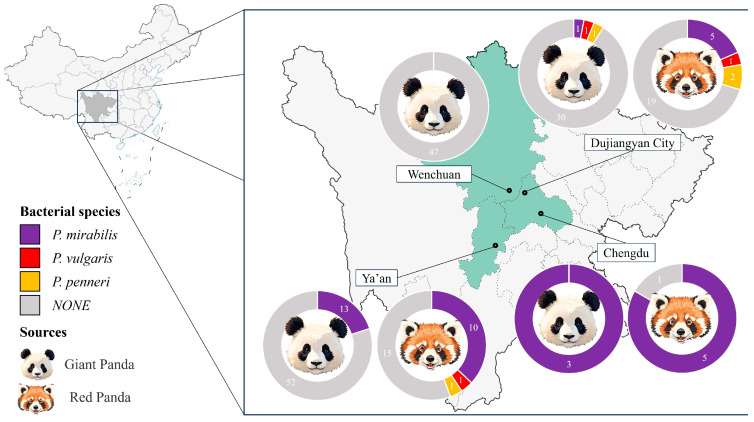
Schematic diagram of the experimental procedure.

A total of 208 fecal samples were collected from captive giant pandas (*Ailuropoda melanoleuca*, *n* = 148) and red pandas (*Ailurus fulgens*, n = 60) housed in zoos and conservation centers across Sichuan Province, with one sample collected per animal. As shown in Figure 1, a total of 37 *Proteus mirabilis* isolates were obtained through streaking on LB and SS agar plates, including 17 from giant pandas (11.5%) and 20 from red pandas (33.3%). The isolation rate was higher in red pandas than in giant pandas.

Regional isolation rates varied, with the highest observed in Chengdu (88.9%, 8/9), followed by Ya’an (25.0%, 23/92) and Dujiangyan (10.0%, 6/60); no *P. mirabilis* isolates were recovered from Wenchuan (0/47). In addition to *P. mirabilis*, three *Proteus vulgaris* and four *Proteus penneri* isolates were also obtained Figure 1.

### 3.2. Genomic Composition and Characteristics of P. mirabilis Isolates from Giant and Red Pandas

Core and pan-genome curves were generated based on gene presence-absence data from 37 *P. mirabilis* strains, as shown in Figure S1. As the number of genomes increased, the size of the core genome steadily decreased and plateaued at 2476 genes, indicating a conserved genetic backbone. In contrast, the pan genome continued to increase, reaching over 5997 genes, suggesting an open and highly variable accessory gene pool across strains.

**Figure 2:**
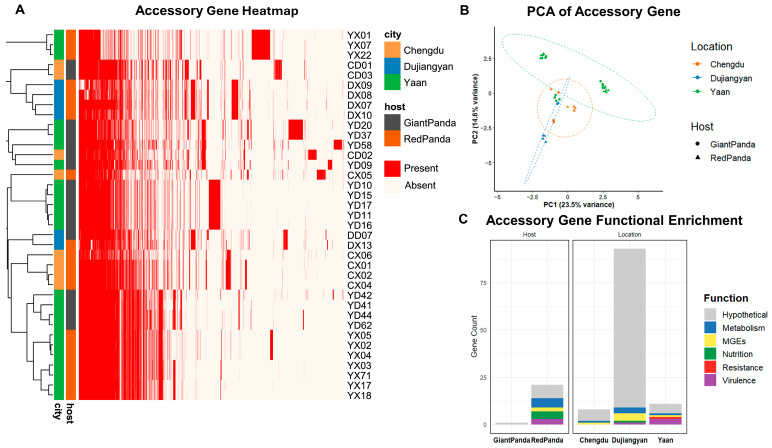
Pan-genome analysis of *Proteus mirabilis* from giant pandas and red pandas. (**A**) Heatmap of variable genes, with red indicating presence and white indicating absence. (**B**) PCA analysis of variable genes, with each point representing a strain. Colors represent the isolation region, and circles represent giant pandas, while triangles represent red pandas. (**C**) Enrichment of variable genes. Bar chart showing the enrichment of genes in each region/host type, with gene functional categories distinguished by color.

Principal component analysis (PCA) based on accessory gene presence/absence (Figure 2B) demonstrated distinct spatial separation among isolates. Giant pandas isolates clustered more tightly, and geographic origin had a greater impact on accessory genome diversity than host species.

### 3.3. Gene Enrichment Analysis

As shown in Figure 2C, enrichment analysis based on gene presence/absence was conducted to explore host- and location-specific features of the accessory genome. *P. mirabilis* strains derived from red pandas exhibited 21 significantly enriched accessory genes, primarily associated with virulence, nutrient acquisition, metabolism, and mobile genetic elements (MGEs), suggesting higher genomic plasticity and adaptive potential. In contrast, only one hypothetical gene was enriched in strains from giant pandas.

A total of 112 location-specific genes were identified, revealing distinct geographic patterns. Dujiangyan isolates harbored the largest number (n = 93), with genes enriched in MGEs and metabolic pathways. Ya’an isolates carried 11 enriched genes, including three virulence factors (*rfaB*, *mxiH*, *hcpA_3*), one resistance gene (*pbpE*), one metabolic gene (*smc*), one MGE *(group_1355*), and five hypothetical proteins. Chengdu isolates exhibited the fewest enriched genes (n = 8), consisting mostly of hypothetical proteins (n = 7) and one MGE (*group_344*). These findings indicate that both host species and geographic origin contribute to shaping the accessory genome composition of *P. mirabilis*, with geographic factors playing a more prominent role.

### 3.4. SNP Analysis

SNP-based phylogenetic analysis revealed distinct clustering patterns shaped by both geographic origin and host species (Figure 4). Strains from Dujiangyan and Chengdu clustered closely in the upper portion of the tree, suggesting shared ancestry or similar transmission dynamics. In contrast, isolates from Ya’an formed a separate cluster, potentially reflecting higher genetic homogeneity or localized selective pressures. Within each geographic group, strains from the same host species tended to cluster together, indicating host-associated constraints on genetic divergence. Collectively, these results highlight the combined influence of geography and host species in shaping the phylogenetic structure of *P. mirabilis*.

### 3.5. Antimicrobial Resistance Profiles of Proteus mirabilis Isolates

**Figure 3:**
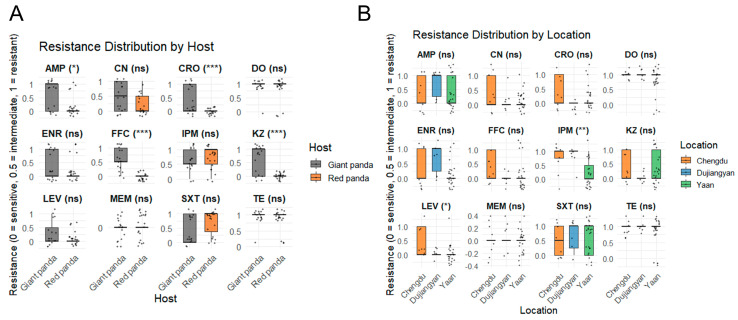
Comparison of antimicrobial resistance levels among *P. mirabilis* isolates from different hosts and locations. (**A**) Resistance distribution by host. Gray boxes represent isolates from giant pandas, and orange boxes represent isolates from red pandas. (**B**) Resistance distribution by geographic location. Orange, blue, and green boxes represent isolates from Chengdu, Dujiangyan, and Ya’an, respectively. Resistance phenotypes are encoded as 0 = sensitive, 0.5 = intermediate, and 1 = resistant. Statistical significance was determined using the Wilcoxon or Kruskal–Wallis test (* *p* < 0.05; ** *p* < 0.01; *** *p* < 0.001; ns = not significant).

Figure 3A summarizes the minimum inhibitory concentration (MIC) profiles of 37 *P. mirabilis* isolates obtained from giant pandas and red pandas, revealing notable differences in antimicrobial resistance patterns between host species. Among the 17 isolates from giant pandas, the highest resistance rates were observed for tetracycline and doxycycline (94.1%, 16/17), followed by ampicillin and cefazolin (58.8%, 10/17). Moderate resistance levels were detected for sulfamethoxazole–trimethoprim and enrofloxacin (41.2%), florfenicol (47.1%), gentamicin and ceftriaxone (35.3%), and imipenem (29.4%). All isolates were fully susceptible to meropenem. In contrast, among the 20 isolates from red pandas, the highest resistance was likewise observed for tetracycline and doxycycline (85.0%, 17/20), followed by sulfamethoxazole–trimethoprim (65.0%) and imipenem (55.0%). Resistance to enrofloxacin (15.0%) and ampicillin (20.0%) was comparatively low, and all isolates were fully susceptible to cefazolin, ceftriaxone, levofloxacin, florfenicol, and meropenem.

Regionally in Figure 3B, isolates from Chengdu and Dujiangyan exhibited significantly higher resistance to imipenem compared to those from Ya’an (*p* < 0.01). Additionally, strains from Chengdu showed greater resistance to levofloxacin relative to isolates from both Dujiangyan and Ya’an (*p* < 0.05).

### 3.6. Distribution and Diversity of Antimicrobial Resistance Genes in Isolated Strains of Proteus mirabilis

**Figure 4:**
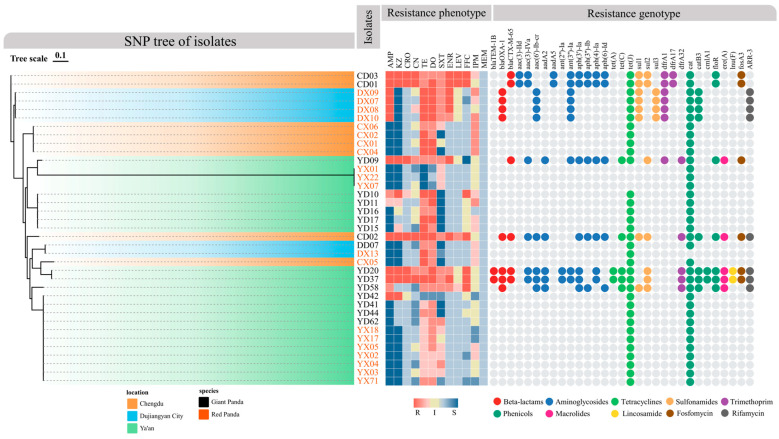
Phylogenetic tree of *P. mirabilis* isolates and their resistance phenotypes and genotypes. The SNP-based phylogenetic tree shows the genetic relationships among isolates. Branch colors represent geographic origin, while isolate name colors indicate host species (black for giant pandas, orange for red pandas). The middle heatmap shows antimicrobial resistance phenotypes: red indicates resistance, yellow indicates intermediate, and blue indicates susceptibility. Darker shades reflect MIC values further from clinical breakpoints. The right panel displays ARGs, with colored dots representing ARG classes: red for β-lactams, blue for aminoglycosides, orange for tetracyclines, purple for trimethoprim, green for phenicols, pink for macrolides, yellow for lincosamides, brown for fosfomycin, and black for rifamycin.

As shown in Figure 4, using the CARD database via Abricate, 31 ARGs belonging to 10 classes—including β-lactams, aminoglycosides, tetracyclines, sulfonamides, trimethoprim, chloramphenicols, macrolides, lincosamides, fosfomycin, and rifamycins—were identified among the 37 *P. mirabilis* isolates. Overall, isolates from giant pandas harbored more ARGs than those from red pandas. Notably, several strains (e.g., YD20, YD37) carried genes spanning nearly all resistance classes, suggesting a high potential for extensive drug resistance (XDR). Of particular concern, the ESBL gene *bla_CTX-M-65_* was exclusively detected in giant panda isolates, suggesting a potential clinical risk. In terms of drug resistance mechanism, aminoglycoside genes were the most prevalent (e.g., *aac(3)-IId*, *aadA5*, *aph(3′)-Ia*, *aph(6)-Id*), followed by β-lactamases *bla_TEM-1B_*, *bla_OXA-1_*, tetracycline resistance genes (*tet(A)*, *tet(C)*, *tet(J)*) and sulfonamide resistance genes (*sul1*, *sul2*, *sul3*).

Regional and host-specific analysis revealed significant variation in ARG distribution. In Chengdu, giant panda isolates had a high ARG load, while red panda isolates carried few. The pattern was reversed in Dujiangyan, where red panda isolates harbored a broader range of ARGs. In Ya’an, strains showed greater variability: some giant panda isolates (e.g., YD20 and YD37) exhibited the highest ARG diversity across all classes, whereas red panda isolates carried very few.

Rare ARGs such as *lnu(F)*, *ere(A)*, *ARR-3*, and *fosA3* were detected sporadically, suggesting limited spread. Collectively, *P. mirabilis* strains exhibited diverse resistance profiles, with giant panda isolates showing greater ARG burden and potential multidrug resistance. ARG distribution was shaped by both host and geographic factors, highlighting the need for targeted regional surveillance.

### 3.7. Correlation Analysis Between ARGs and Drug Resistance Phenotypes

The resistance gene profiles generally aligned with the observed phenotypic resistance patterns (Figure 4). Among β-lactam-resistant strains, 78.6% carried corresponding resistance genes, and all ceftriaxone-resistant isolates carried the *bla_CTX-M-65_* gene. Similarly, resistance to gentamicin, sulfonamides, and trimethoprim consistently correlated with the presence of their respective ARGs. Strains carrying *aac(6′)-Ib-cr* exhibited resistance or intermediate susceptibility to enrofloxacin and levofloxacin.

Although nearly all isolates carried the cat gene, florfenicol resistance was observed only in those harboring additional chloramphenicol resistance genes (*catB3*, *cmlA1*, *floR*). In addition, previous studies have shown that *P. mirabilis* exhibits intrinsically elevated MIC values to imipenem; thus, many strains displayed resistance or intermediate susceptibility without harboring known carbapenemase genes.

### 3.8. Virulence-Associated Traits of Proteus mirabilis Isolated Strains

As shown in Figure S2, *P. mirabilis* strains isolated from giant pandas generally exhibited stronger biofilm-forming abilities than those from red pandas. Based on the ODc, the biofilm-forming ability of isolates can be divided into the following four types: non-biofilm formers (OD ≤ ODc), weak (ODc < OD_590_ ≤ 2ODc), moderate (2ODc < OD_590_ ≤ 4ODc), strong (4ODc < OD_590_ ≤ 8ODc), and very strong (OD_590_ > 8ODc). Quantitative analysis revealed significantly higher biofilm biomass among giant panda-derived strains (OD_595_: 1.03–2.77) compared to red panda-derived strains (OD_595_: 0.865–1.78), with this difference being statistically significant (t = 8.45, df = 89.34, *p* = 4.94 × 10^−13^). Regarding swarming motility, all giant panda isolates and most red panda isolates displayed strong motility, while three red panda strains (YX05, YX71, DX13) exhibited only moderate motility.

Significant differences in urease activity were also observed between the two host groups. Urease activity among giant panda-derived strains ranged from 0.0093 to 0.0146 U/10^6^ cells (mean = 0.0120, SD = 0.00241), while red panda strains showed a broader range (0.0103–0.0244 U/10^6^ cells) and a higher average activity (mean = 0.0159, SD = 0.00485). This difference was statistically significant (t = –5.62, df = 85.06, *p* = 2.41 × 10^−7^), suggesting host-associated differences in urease expression (Figure S3). All 37 isolates exhibited α-hemolysis, as indicated by uniform translucency on 5% sheep blood agar plates, likely resulting from their strong swarming motility (Figure S4).

### 3.9. VAGs Analysis of Isolated Strains

**Figure 5:**
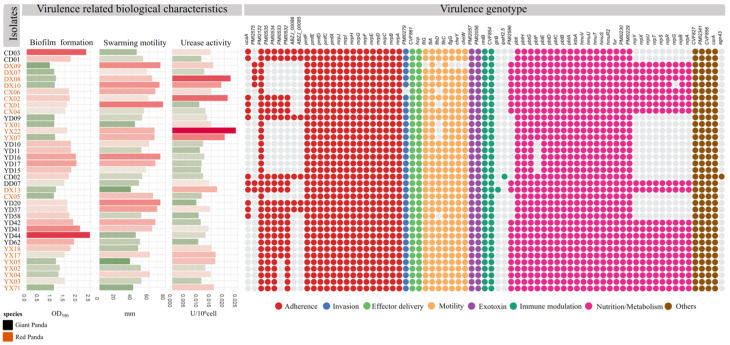
Virulence-related biological characteristics and virulence gene profiles of *P. mirabilis* isolates. The left panel shows the results of virulence-associated phenotypic assays (biofilm formation, motility, and urease activity). The right heatmap displays the presence or absence of virulence genes across isolates. Colored dots indicate the functional categories of virulence genes.

As shown in Figure 5, a total of 73 VAGs were identified across the 37 *P. mirabilis* isolates, encompassing functions related to motility, chemotaxis, toxin production, adhesion, and immune evasion. Most isolates carried more than 60 VAGs, with 44 genes conserved across all strains, forming a core virulence repertoire shared by both giant panda- and red panda-derived isolates. Variation at the strain level was observed. The red panda-derived strain DX13 harbored the highest number of VAGs (n = 69), although on average, isolates from giant pandas carried slightly more virulence genes than those from red pandas. Some genes—such as *agn43*, *ipaH2.5*, and *gtrB*—were found in only a few isolates, suggesting potential acquisition via horizontal gene transfer or niche-specific adaptation.

Among the 24 adhesion-related genes, 16 were universally present. Notably, seven genes encoding uroepithelial cell adhesins (UCA) were detected in 54.8% of strains, with DD07 and DX13 harboring all seven. Capsule-associated genes (*ABZJ_00085* and *ABZJ_00086*) were exclusively found in giant panda-derived strains. All isolates carried key biofilm-associated genes (*PmfACDEF*, *MR/P fimbriae*) and hemolysin genes (*hpmA*, *hpmB*). Only one invasion gene, *ZapA*, was identified. Flagellar genes were largely conserved across strains, although DX09 possessed only two, and a few isolates lacked *flhD*, a transcriptional activator of flagellar biosynthesis. Immune-related genes (*rmlB*, *CVF854*) were universally present, whereas *gtrB* and *ipaH2.5* were rare. Genes involved in nutrient acquisition, such as the siderophore systems *pbtABCDEFGHI*, *hmuR2STUV*, and *fur*, were widely distributed. Non-ribosomal peptide synthetase (*NRPS*) gene clusters for iron uptake were complete in 20 isolates, while others lacked them entirely. Finally, all strains harbored *csrA*, a key global regulator involved in motility and biofilm formation.

### 3.10. Correlation Analysis Between VAGs and Phenotypic Traits

**Figure 6:**
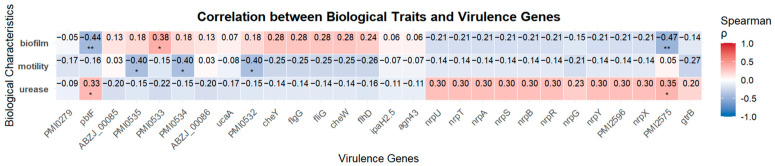
Spearman’s correlation heatmap between *P. mirabilis* biological characteristics and virulence genes. Each cell indicates the Spearman correlation coefficient (ρ) between the trait and gene presence. Positive correlations are shown in red, and negative correlations in blue. Asterisks denote statistically significant correlations (* *p* < 0.05; ** *p* < 0.01).

As shown in Figure 6, no strong correlations were observed between VAGs and phenotypic traits. However, weak positive correlations were identified between *PMI0533* and biofilm formation (r = 0.38, *p* < 0.05), as well as between *pbtF* or *PMI2575* and urease activity (r = 0.33 and 0.35, respectively; *p* < 0.05). Meanwhile, *PMI0532*, *PMI0534*, and *PMI0535* showed moderate negative correlations with swarming motility (r = −0.40, *p* < 0.05), and *pbtF* and *PMI2575* were also negatively associated with biofilm formation (r = −0.44 and −0.47, respectively; *p* < 0.01).

### 3.11. Correlation Between Mobile Genetic Elements (MGEs) and ARGs/VAGs

**Figure 7:**
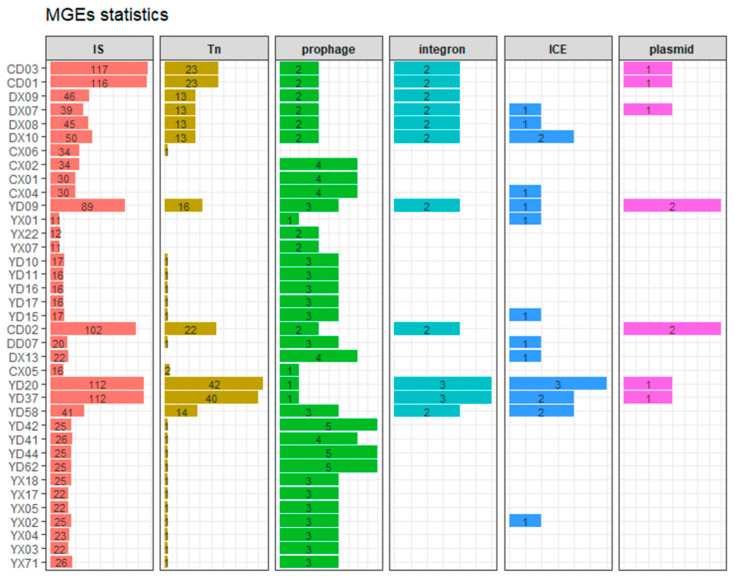
Stacked column charts showing the number of six types of mobile genetic elements (MGEs) detected in each *P. mirabilis* strain. Each panel represents a distinct MGE type, including insertion sequences (IS), transposons (Tn), prophages, integrons, integrative and conjugative elements (ICEs), and plasmids. The horizontal bars represent the counts of each MGE type in individual strains. Strain IDs are labeled on the y-axis, and the x-axis denotes the number of elements. Bars are color-coded by MGE type.

In this study, a total of 1855 mobile genetic elements (MGEs) were identified, including 1437 insertion sequences (IS), 252 transposons (Tn), 24 integrons, 9 plasmids, 102 prophages, and 31 integrative and conjugative elements (ICEs), as shown in Figure 7. Strains from CD01-03, YD20, and YD37 carried more than 100 IS elements and over 20 Tn elements, showing higher levels of antimicrobial resistance. This trend was particularly evident for integrons and plasmids, as all strains carrying integrons and plasmids were MDR.

**Figure 8:**
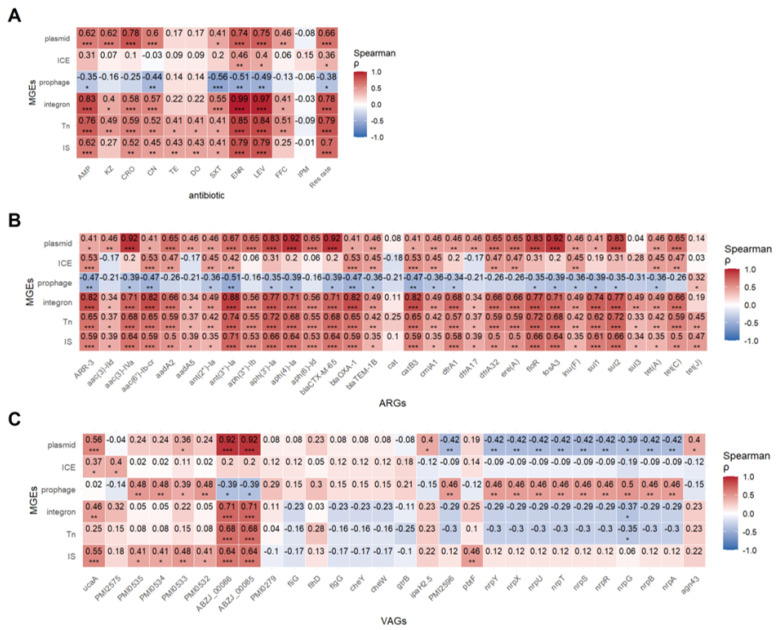
Spearman correlation between MGEs and resistance phenotype, ARGs and virulence VAGs. (**A**) Spearman’s correlation heatmap between MGEs and resistance phenotype. (**B**) Spearman’s correlation heatmap between MGEs and ARGs. (**C**) Spearman’s correlation heatmap between MGEs and VAGs. Each cell indicates the Spearman correlation coefficient (ρ) between the trait and gene presence. Positive correlations are shown in red, and negative correlations in blue, with intensity proportional to the strength of the correlation (see color bar). Asterisks denote statistically significant correlations (* *p* < 0.05; ** *p* < 0.01; *** *p* < 0.001).

Spearman correlation analysis revealed that insertion sequences (IS), transposons (Tn), integrons, and plasmids were positively associated with resistance to all Antimicrobials except imipenem. ICEs correlated positively with enoxacin and levofloxacin resistance, while prophages showed negative correlations with resistance to five Antimicrobials, suggesting they may suppress resistance (Figure 8A).

The correlation between MGEs and resistance genes was also strong (Figure 8B). Except for the cat gene, all 30 resistance genes were positively correlated with IS and Tn to varying degrees (r = 0.33–0.74, *p* < 0.05–0.001). Integrons were positively correlated with 29 resistance genes, with the strongest correlation observed for ant(3″)-Ia (r = 0.88, *p* < 0.001). ICEs exhibited negative correlations with *aac(3)-IId*, *aadA5*, *CAT*, and *dfrA1*, while showing positive correlations with other resistance genes. Plasmids were positively correlated with nearly all resistance genes except *cat*, *sul3*, and *tet(J)*. Interestingly, ICEs and plasmids showed opposing patterns, hinting at gene-specific MGE preferences. Prophage-gene correlations echoed resistance profiles.

Correlation between MGEs and virulence genes is shown in Figure 8C. IS elements were significantly positively correlated with adhesion and iron acquisition genes (*p* < 0.05). Plasmids significantly positively correlated with *ABZJ*_*00085* and *ABZJ*_*00086* (*p* < 0.001), but negatively with nutrient acquisition genes, indicating that plasmids might negatively impact bacterial fitness. Prophages were positively correlated with adhesion and nutrient genes (*p* < 0.05) but negatively associated with *ABZJ*_*00085*/*00086*, highlighting their complex role in virulence *(p* < 0.05).

### 3.12. Co-Localization of Mobile Genetic Elements (MGEs) with ARGs and VAGs

**Figure 9:**
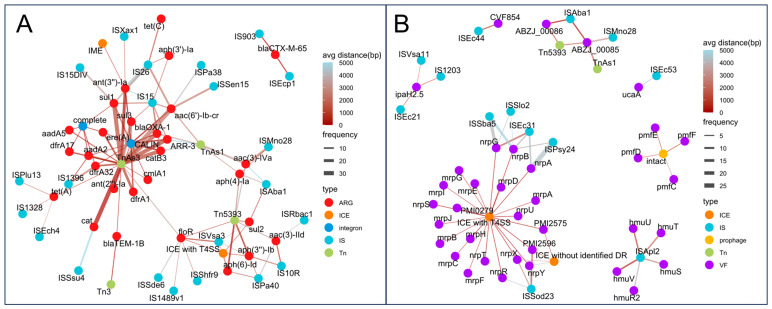
Co-localization networks between MGEs and resistance or virulence genes. (**A**) Co-localization network of ARGs and MGEs. (**B**) Co-localization network of VAGs and MGEs. Each node represents a genetic element (ARG, VAG, or MGE), colored by type. Edges indicate co-localization events, with edge width proportional to frequency and edge color indicating average distance (from red = close, blue = distant).

To further assess the link between MGEs and ARGs/VAGs, genes located within 5000 bp of an MGE or fully contained within it were considered co-localized. As shown in Figure 9A, several IS elements, such as *IS903* and *ISEcp1,* consistently co-localized with *bla_CTX-M-65_*, while *IS15*, *IS26*, and *ISAba1* were associated with multiple ARGs, suggesting these resistance genes may form resistance islands and have the potential for horizontal transfer.

*Tn5393* showed frequent co-localization with various aminoglycoside and sulfonamide resistance genes. CALINS and complete integrons carried a broad spectrum of ARGs across nearly all Antimicrobial classes. Moreover, ICEs with type IV secretion systems (T4SS) also carried resistance genes such as *aph(3″)-Ib*, *aph(6)-Id*, *floR*, and *sul2*. Additionally, we identified an Integrative and Mobilizable Element (IME) carrying *ant(3″)-Ia*. In terms of VAGs, extensive co-localization was also observed (Figure 9B). Some IS elements were co-localized with specific VAGs, such as *IS1203* with *ipaH2.5*. More commonly, an IS element was co-localized with multiple VAGs, such as *ISApl2* with *hmuSTUVR2*; *ISSod23* with *PMI2596* and *nrpRXY*; and *ISEc31* with *nrpABG*. For Tn elements, *Tn5393* was co-localized with *ABZJ_00085* and *ABZJ_00086*, and *TnAs1* was co-localized with *ABZJ_00085*. Furthermore, ICEs with T4SS carried numerous VAGs, including *nrpABGRSTUXY*, *mrpABCDEFGHIJ*, *PMI2575*, *PMI2596*, and *PMI0279*. Notably, some prophages were co-localized with VAGs, such as *pmfC*, *pmfD*, *pmfE*, and *pmfF.*

These findings support the idea that ISs, Tns, ICEs, and integrons facilitate the horizontal transfer of both resistance and virulence genes, while prophages mainly contribute to virulence gene mobility.

### 3.13. Plasmids and ICEs Carrying ARGs and VAGs in P. mirabilis Isolates

Plasmids and integrative and conjugative elements (ICEs) are key mobile genetic elements (MGEs) that enable the horizontal transfer of ARGs and VAGs. In this study, 9 plasmids and 18 ICEs (including ICEs lacking T4SS, i.e., IMEs) were identified in 16 *P. mirabilis* strains. Except for DD07 and YX02, all MGE-positive strains were multidrug-resistant (MDR).

**Figure 10:**
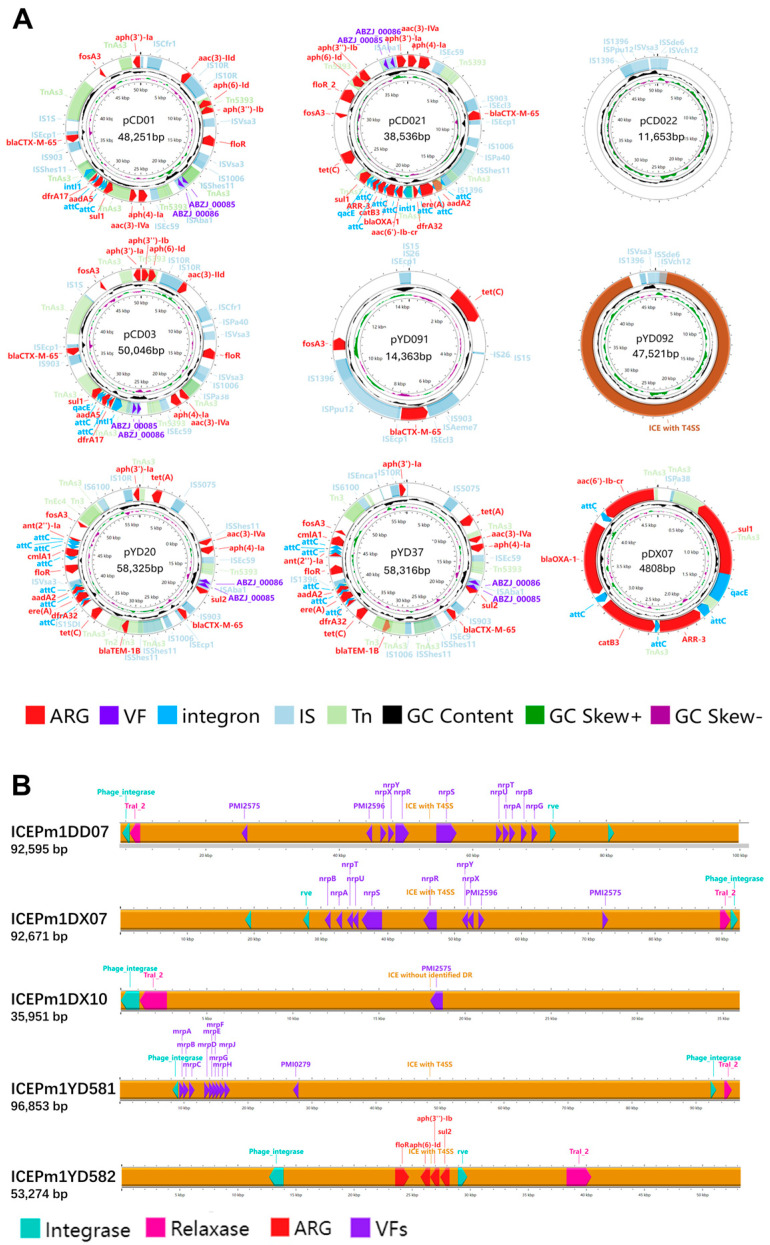
(**A**) Circular maps of plasmids and integrative elements in *P. mirabilis* isolates. Arrows indicate genes on the forward strand (strand = “+”, clockwise). Features are color-coded: red for ARGs, purple for VFs, blue for integrons, light blue for ISs, light green for Tns. Inner rings show GC content (black) and GC skew (green for +, purple for –). (**B**) Linear maps of ICE elements in *P. mirabilis* isolates. Arrows indicate genes on the forward strand (strand = “+”, pointing to the right). Features are color-coded: red for antimicrobial resistance genes (ARGs), purple for virulence factors (VFs), pink for relaxase, and light blue for integrase.

As shown in Figure 10A, 3 plasmids carried complete integrons containing intI, and another 3 harbored CALINs; 7 plasmids contained ARGs, with 5 carrying over 10 genes. The plasmid pCD021 encoded 17 ARGs across various antimicrobial classes, including β-lactams (ESBLs), aminoglycosides, tetracyclines, and others. Notably, *bla_CTX-M-65_* appeared on 5 plasmids, and multiple ARGs were embedded within integrons, posing a high HGT risk. These ARG-rich plasmids were mostly from Chengdu and Ya’an giant panda isolates. Virulence genes *ABZJ*_*00085*/*86* were also frequently detected on these plasmids. Plasmid pCD022 lacked known ARGs/VAGs, suggesting the presence of unknown functional genes. As shown in Figure 10B, 5 of the 18 ICEs (*SXT*/*R391* family) carried ARGs or VAGs. Only *ICEPm1YD582* carried ARGs, while others carried virulence genes related to adherence or nutrient metabolism. Plasmids were mainly found in Chengdu and Ya’an isolates, especially from giant pandas, while ICEs were more common in strains from Ya’an and Dujiangyan, without strong host preference.

These findings highlight the regional and host-related variation in MGE distribution and their contribution to resistance and virulence gene dissemination.

## 4. Discussion

Captive giant pandas and red pandas are commonly infected by *Proteus mirabilis*, yet data on their resistance and gene transmission risks are scarce. This limits informed antimicrobial use and raises concerns over MDR and potential zoonotic transmission [8]. In this study, *P. mirabilis* strains were isolated from giant panda and red panda feces in Sichuan, China, and analyzed for antimicrobial resistance, virulence, and gene mobility. Pangenome analysis revealed host- and region-specific patterns, highlighting transmission risks. These findings offer critical insights for managing *P. mirabilis* infections and monitoring resistance spread in wildlife.

In this study, 37 *P. mirabilis* strains were isolated from 208 fecal samples of captive giant pandas and red pandas in Sichuan. The isolation rate was significantly higher in red pandas (33.3%) than in giant pandas (11.5%), indicating host-specific colonization patterns. This may relate to differences in gut microbiota, which warrant further 16S rRNA analysis [28]. No strains were recovered from Wenchuan, possibly due to limited sample size or low local colonization, suggesting ecological factors also influence distribution.

Regionally, the highest isolation rate was observed in Chengdu (88.9%, 8/9), followed by Ya’an (25.0%, 23/92) and Dujiangyan (10.0%, 6/60), while no *P. mirabilis* isolates were recovered from Wenchuan (0/47). Notably, the sampling site in Chengdu is located in an urban center, whereas the sites in Ya’an and Dujiangyan are situated in suburban areas, and the site in Wenchuan is located within a national nature reserve. A positive correlation was observed between the level of human activity around the sampling sites and the isolation rate of *P. mirabilis*, suggesting that increased anthropogenic disturbance may facilitate the transmission of this bacterium.

The growing antimicrobial resistance of *P. mirabilis*, including XDR and PDR strains, has become a public health concern [7]. Broth dilution remains one of the most widely used methods to determine MICs for AMR assessment [29]. The results of the broth microdilution assay showed that the drug resistance rates of the isolates in this study ranged from 0 to 89.2%. Strains from giant pandas showed significantly higher resistance than those from red pandas, with 53% being MDR and several classified as XDR. This may result from higher antimicrobial exposure or environmental accumulation in captive giant pandas. Conversely, red panda strains showed lower resistance, likely reflecting differences in host microecology and husbandry. As red pandas experience illness less frequently, they are generally administered fewer antibiotics, which may contribute to the lower resistance observed in their isolates. Notably, giant panda isolates had significantly higher resistance to tetracyclines, penicillins, and cephalosporins (e.g., cefazolin, ceftriaxone; *p* < 0.05), suggesting host-driven resistance evolution. Compared with prior reports, giant panda strains exhibited intermediate resistance levels between pig and dog-derived isolates, while red panda strains had the lowest resistance overall [13,30].

A key contributor to bacterial resistance is the presence of ARGs [31]. Using the CARD database, we identified ARGs in *P. mirabilis* isolates, which largely matched phenotypic resistance profiles [18], particularly for β-lactams, aminoglycosides, sulfonamides, and tetracyclines. β-lactamase-mediated resistance has become increasingly prevalent in *P. mirabilis*, and in this study, we identified three β-lactamase genes: *bla_TEM-1B_*, *bla_OXA-1_*, and *bla_CTX-M-65_* [32]. ESBL-producing strains represent a major global health concern, significantly increasing morbidity and mortality in hospitalized patients [33]. Notably, the ESBL gene *bla_CTX-M-65_* was found only in giant panda strains, indicating a potential clinical concern. Although quinolones are considered effective alternatives to β-lactams, resistance was detected in 27% of isolates, rising to 41.2% in giant panda strains [34]. The plasmid-mediated gene *aac(6′)-Ib-cr*, associated with reduced susceptibility to both aminoglycosides and quinolones, was identified in both host species. Resistance to fosfomycin—an older antimicrobial now repurposed for MDR infections—was also observed via the *fosA3* gene in giant panda isolates [35]. Additional resistance genes related to chloramphenicols, sulfonamides, rifamycins, and macrolides were also detected. Combined with the intrinsic resistance of *P. mirabilis*, these results underscore serious clinical challenges. In terms of species, giant panda isolates carried significantly more ARGs than those from red pandas, with strains like CD01 and CD02 harboring nearly all major resistance classes. Overall, red panda strains had low ARG abundance across all regions. Geographically, and Chengdu-derived isolates showed the highest ARG diversity. This host- and region-specific pattern suggests localized ARG accumulation or host-specific enrichment, potentially driven by anthropogenic antimicrobial pressure. These findings emphasize the need for targeted surveillance and antimicrobial stewardship in high-risk areas.

Biofilm formation and swarming motility are key virulence traits in *P. mirabilis*, facilitating epithelial adherence and rapid migration, particularly in urinary tract infections [3], and Biofilm formation is a well-established mechanism for antimicrobial tolerance [36]. In this study, strains isolated from both giant and red pandas exhibited stronger biofilm formation and motility than pig- and dog-derived strains reported previously [13,29], possibly due to host-specific selective pressures or the pandas’ low gut microbial diversity, which may favor rapid *P. mirabilis* colonization [37]. This suggests a non-negligible infection risk in panda hosts. A total of 73 VAGs were identified, with most isolates harboring over 60, indicating high pathogenic potential. Core VAGs—including *hpmA/B* (hemolysins), *mrpABCDEFGHIJ* (adhesion), *csrA* (global regulation), and siderophore-related genes like *pbt* and *fur*—were consistently present across all strains, suggesting their essential role in maintaining virulence. Certain strains (e.g., *DX13*, *DD07*) exhibited notably high VAG loads. Host- and region-specific VAGs such as *ABZJ_00085/00086* (giant panda-specific) and *ipaH2.5* (unique to strain CD02) may reflect ecological or host-driven acquisition of novel virulence elements. Correlation analysis showed weak associations between most VAGs and phenotypic traits, indicating functional redundancy or synergistic effects that warrant further transcriptomic or proteomic investigation. Importantly, eight previously unreported VAGs in *P. mirabilis* were identified—*ABZJ_00085*, *ABZJ_00086*, *CVF861*, *CVF854*, *gtrB*, *fur*, *CVF827*, and *agn43*—involved in adherence, immune evasion, effector delivery, and nutrient acquisition. Several of these genes were co-localized with MGEs, suggesting acquisition via horizontal gene transfer. These findings expand the known virulence repertoire of *P. mirabilis* and point to potential host-specific adaptation mechanisms in the panda gut.

We also examined ARG–MGE co-localization. ARGs located within 5 kb of MGEs were classified as potentially mobile [38]. The analysis revealed that MGEs play a central role in mediating the horizontal transfer of ARGs and VAGs. Specific IS elements such as *IS903* and *ISEcp1* were frequently co-localized with *bla_CTX-M-65_*. Other IS elements (e.g., *IS15*, *IS26*) showed frequent co-localization with multiple resistance genes, suggesting their role in forming resistance islands and promoting ARG co-transfer. *Tn5393* was frequently co-localized with aminoglycoside resistance genes, indicating a central role in spreading this resistance type. CALINs and integrons were also key structures, carrying ARGs via integrase-independent and -dependent mechanisms, respectively [39]. For VAGs, co-localization with MGEs was also prominent. Several IS elements (e.g., *ISApl2*, *ISEc31*) and Tn elements were co-localized with VAGs, while ICEs—particularly those encoding T4SS frequently harbored complete virulence clusters such as mrp and nrp, suggesting enhanced adhesion and metabolic adaptation. Some prophages were co-localized with pmf-related genes, indicating their potential role in virulence evolution despite not carrying ARGs.

The analysis of plasmids and ICEs highlighted their critical role in HGT-mediated dissemination of ARGs and VAGs, owing to their ability to replicate autonomously and transfer between bacterial hosts [40,41]. Multiple plasmids were found to carry complete integrons or CALINs, enhancing their capacity to acquire and disseminate ARGs. Notably, five plasmids harbored more than ten ARGs each, with pCD021 carrying the highest number—17 ARGs. These genes conferred resistance to a broad spectrum of antimicrobial classes, including β-lactams, aminoglycosides, tetracyclines, sulfonamides, trimethoprim, phenicols, macrolides, fosfomycin, and rifamycins. More importantly, the extended-spectrum β-lactamase (ESBL) gene *bla_CTX-M-65,_* which confers resistance to third-generation cephalosporins, was detected on five plasmids. The presence of this clinically significant resistance gene in wildlife-associated strains poses a substantial challenge to antimicrobial therapy [33]. Of particular concern, plasmids also carried the immune evasion genes *ABZJ_00085* and *ABZJ_00086*, which may synergistically enhance both pathogenicity and persistence [42]. Regionally, plasmids were more commonly found in giant panda strains from Chengdu and Ya’an, while ICEs were more broadly distributed across regions and hosts. This indicates that the dissemination of MGEs is influenced by both ecological and host-specific factors, emphasizing the importance of surveillance in wildlife populations and their habitats. Collectively, these findings demonstrate the widespread co-localization and dissemination potential of ARGs and VAGs via MGEs in *P. mirabilis* from captive pandas, raising significant concerns about zoonotic transmission, resistance evolution, and public health risks.

## 5. Conclusions

This study systematically investigated the antimicrobial resistance, virulence traits, and MGE-mediated gene dissemination potential of *P. mirabilis* from captive giant pandas and red pandas in Sichuan, China. Although the isolation rate was higher in red pandas, strains from giant pandas showed significantly greater resistance and virulence. Some isolates exhibited multidrug or extensive drug resistance and carried numerous core and novel VAGs, indicating high pathogenic potential. Genotypic profiles of ARGs and VAGs closely matched phenotypic data and displayed clear host- and region-specific patterns. In addition, MGEs played a key role in the horizontal transfer of ARGs and VAGs, with certain plasmids and ICEs co-carrying multiple resistance and virulence genes. In summary, our study offers valuable insights into the evolution of AMR and public health risks associated with *P. mirabilis* isolated from captive giant pandas and red pandas. It provides a scientific foundation for advancing clinical prevention and control strategies, as well as for further exploration of resistance and pathogenic mechanisms.

## Data Availability

The whole genome sequence data of 37 *Proteus mirabilis* isolated strains have been submitted to the NCBI (https://www.ncbi.nlm.nih.gov/) under the BioProject accession number: PRJNA1274410.

## Supplementary Materials

The following supporting information can be downloaded from https://www.mdpi.com/article/10.3390/microorganisms13081802/s1: Figure S1. Core and pan genome curves of *Proteus mirabilis* isolates; Figure S2. Biofilm formation ability of the isolated strains of *Proteus mirabilis*. All the results are represented as mean ± SD; Figure S3. Urease activity of the isolated strains of *Proteus mirabilis*. All the results are represented as mean ± SD; Figure S4. Hemolytic activity of the isolated strains of *Proteus mirabilis.*
