# Supplementary material for: Proteus mirabilis from Captive Giant Pandas and Red Pandas Carries Diverse Antimicrobial Resistance Genes and Virulence Genes Associated with Mobile Genetic Elements
Source: Microorganisms. 2025 Aug 1;13(8):1802. doi: 10.3390/microorganisms13081802 (PMC12388390; doi:10.3390/microorganisms13081802)
Supplement: Supplementary file 1 [file microorganisms-13-01802-s001.zip › microorganisms-3730435-supplementary.pdf]

Supplementary Materials

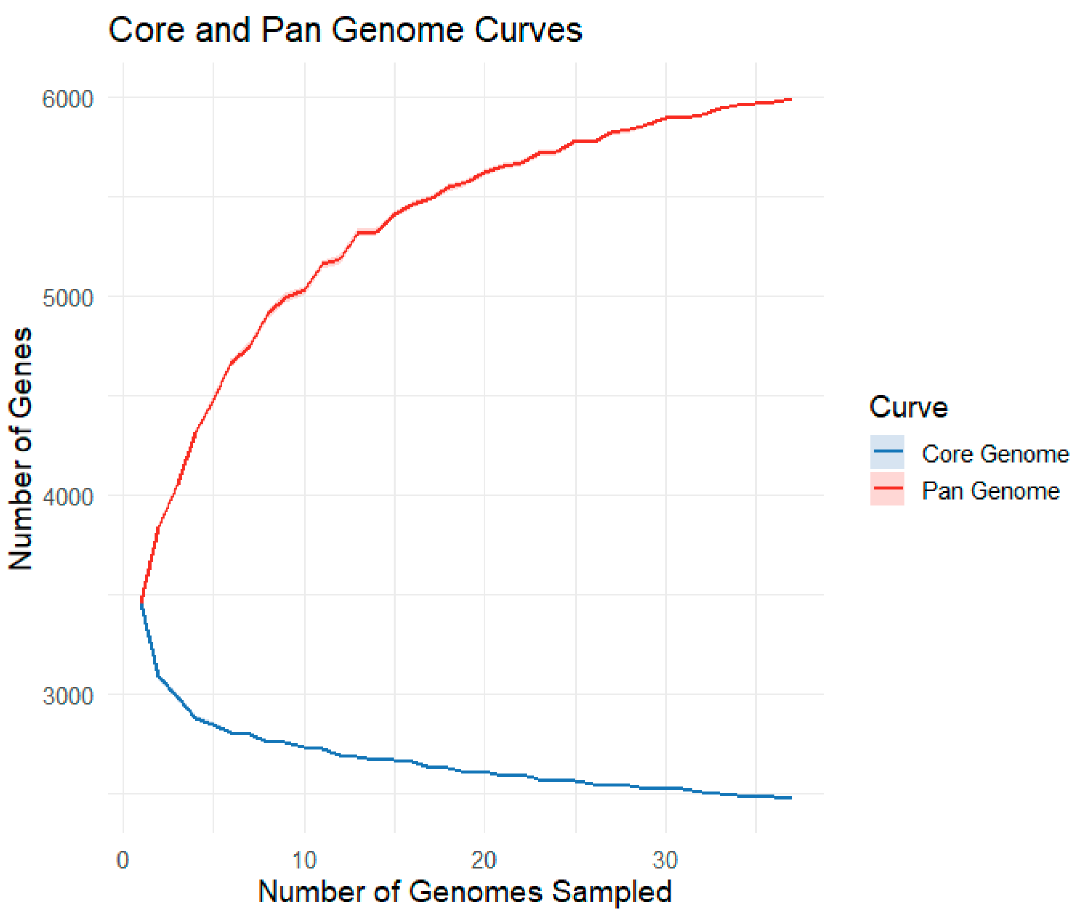

Figure S1. Core and pan genome curves of Proteus mirabilis isolates.

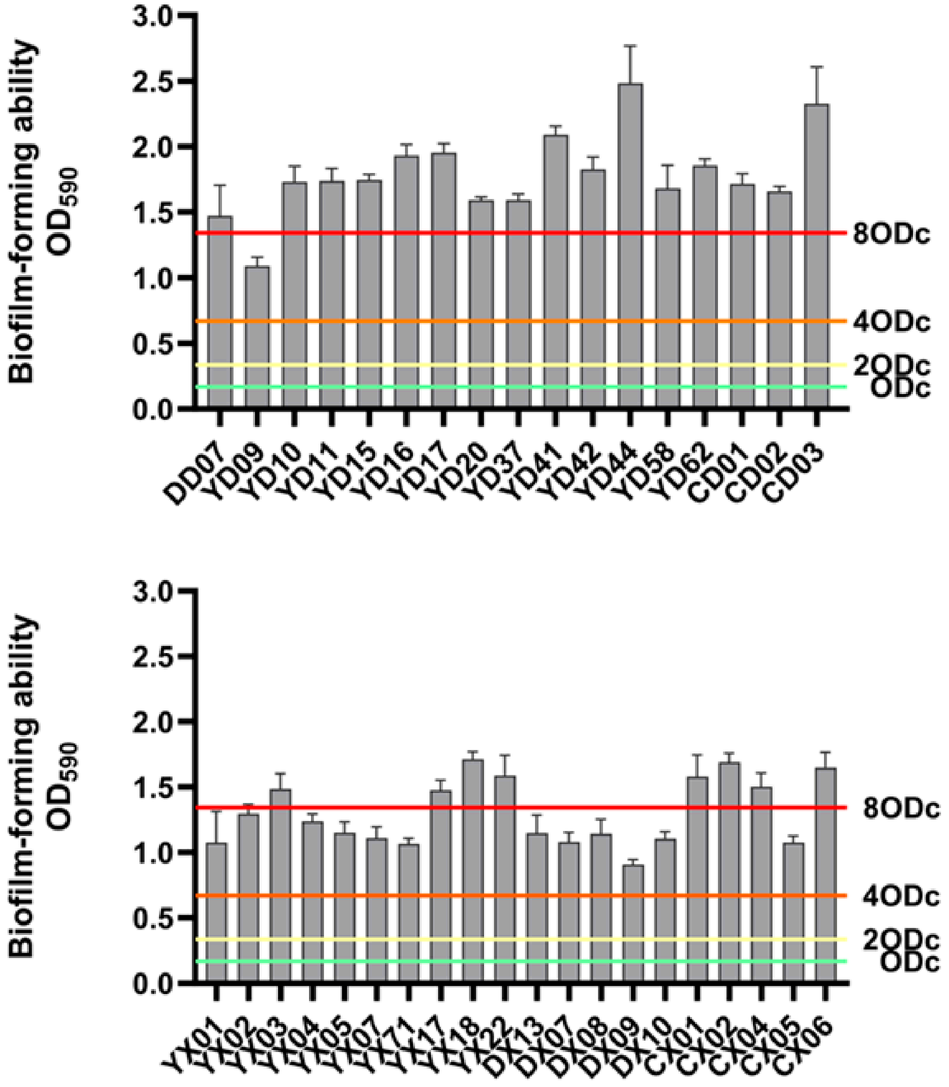

Figure S2. Biofilm formation ability of the isolated strains of Proteus mirabilis. All the results are represented as mean ± SD.

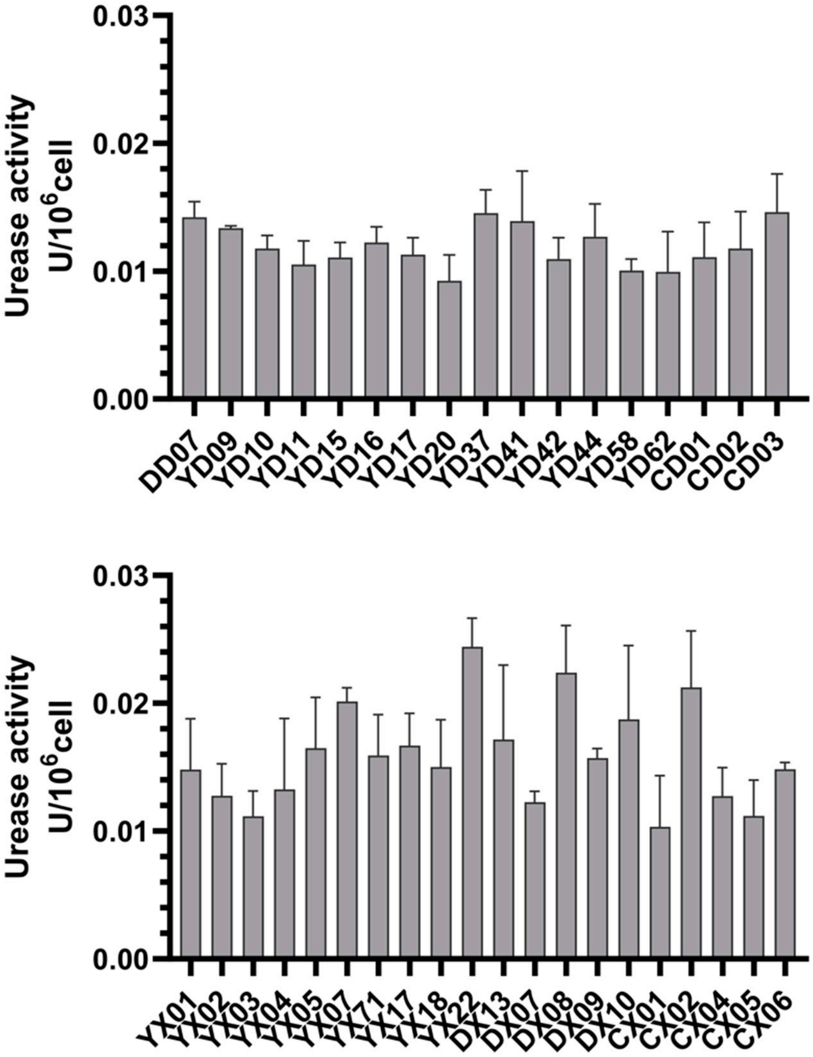

Figure S3. Urease activity of the isolated strains of Proteus mirabilis. All the results are represented as mean ± SD.

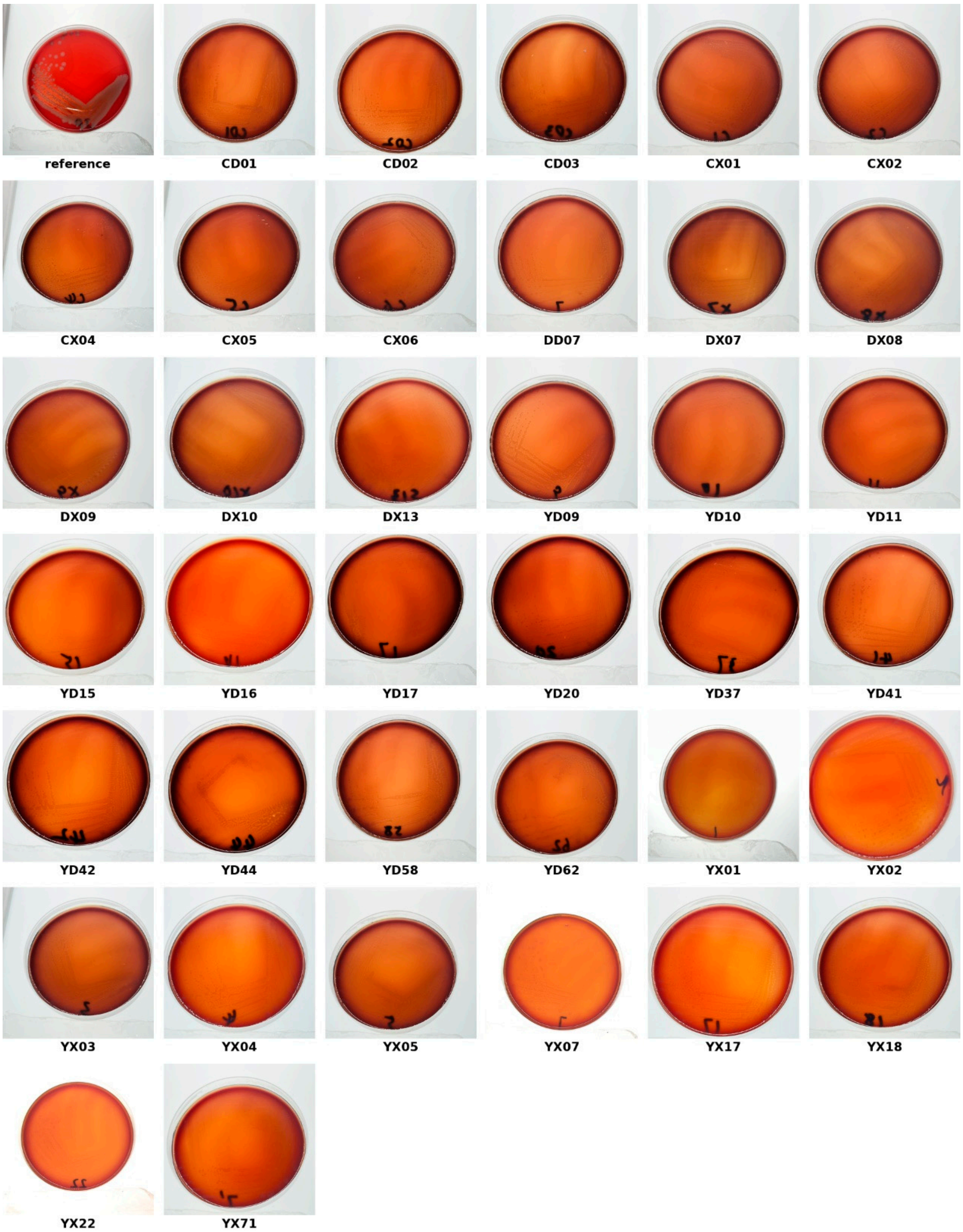

Figure S4. Hemolytic activity of the isolated strains of Proteus mirabilis.
